# A Critical Review on the Role of Food and Nutrition in the Energy Balance

**DOI:** 10.3390/nu12041161

**Published:** 2020-04-22

**Authors:** Simona Bo, Maurizio Fadda, Debora Fedele, Marianna Pellegrini, Ezio Ghigo, Nicoletta Pellegrini

**Affiliations:** 1 Department of Medical Sciences, University of Turin, 10126 Turin, Italy; mariannapellegrini87@gmail.com (M.P.); ezio.ghigo@unito.it (E.G.); 2 Dietetic and Clinical Nutrition Unit, S. Giovanni Battista Hospital, Città della Salute e della Scienza, 10126 Turin, Italy; maurizio.fadda@unito.it (M.F.); d.fedele85@gmail.com (D.F.); 3 Department of Agricultural, Food, Environmental and Animal Sciences, University of Udine, 33100 Udine, Italy; nicoletta.pellegrini@uniud.it

**Keywords:** energy expenditure, energy balance, obesity, fat burners

## Abstract

The mass media has increasingly frequently suggested to the general population that specific foods or nutritional schemes are able to affect both human metabolism and energy expenditure, thus facilitating weight loss. This critical review is aimed at assessing available evidence on the roles of nutrients, food and dietary regimens in energy intake and energy expenditure. We queried the National Library of Medicine, the Cochrane Library, Excerpta Medica dataBASEand the Cumulative Index to Nursing and Allied Health Literature database, and a search strategy was performed by using database-specific subject headings and keywords. We found that available scientific evidence on these topics is scarce, and that the limited number of available studies often have poor methodological quality. Only a few foods show beneficial effects on metabolism and energy expenditure, as the human energy balance is complex and multifactorial. Finally, microbiota may interfere with the intake, use and expenditure of energy in the human body. Conclusive evidence is still lacking, and, at present, it is not possible to identify a food or a diet with a significant impact on human energy expenditure.

## 1. Introduction

A large amount of misleading news has circulated on social media, blogs, TV and magazines about human nutrition. A specific food or nutrient is often presented as a cure for one or more pathologies, ranging from diabetes mellitus to cancer or Alzheimer's disease [1]. A great amount of information without scientific reliability relative to the treatment of overweightness/obesity is available, a topic in which myths and presumptions are very common [2]. Comprehension of the individual energy balance is particularly complex, owing to physiological compensation to changes in energy intake and/or expenditure [3]. Social media, the Internet, TV and magazines frequently propose direct-to-consumer “information” about food, dietary schemes or supplements which increase the energy expenditure and/or burn fats or, otherwise, reduce the energy expenditure and lead to fat accumulation. However, most of these advertisements contain mis- or dis-information. Some examples include: “drink a lot and consume fat-burning foods” (e.g., pineapple, ginger, onion, avocado, asparagus, celery, chili, broccoli, green tea, garlic, etc.) and “avoid the foods that make you fat” (e.g., pasta, bread and foods containing gluten, oil, dairy products, etc.), in order to lose weight [4]. All these suggestions are generally incorrect: there are no foods with negative calories and focusing on one or a few foods or nutrients does not work, as a multifaceted and individualized program with careful follow-up over time is required to lose weight [5]. This kind of mis-/dis-information is particularly concerning, owing to its influence on the general population, and such wrong beliefs have been found to be hard to correct, especially in people with lower cognitive ability [6].

The aim of the present paper is to critically review the available evidence about the roles of nutrients, food, and dietary regimens on energy intake and energy expenditure, taking into consideration all the conditions potentially impacting on the final energy balance, including the gut microbiota. In particular, we analyzed the following topics:

(i) The energy balance in humans;
(ii) Energy intake from food;
(iii) Energy expenditure due to food intake;
  - the role of nutrients;
  - the role of foods;
  - the role of diet plans;
(iv) The impact of the gut microbiota on the human energy balance.

## 2. Methods

The following databases were queried: PubMed (National Library of Medicine), the Cochrane Library, Excerpta Medica dataBASE (EMBASE) and the Cumulative Index to Nursing and Allied Health Literature (CINAHL). The search strategy was performed by using database-specific subject headings and keywords (i.e., energy, energy expenditure, energy balance, energy intake, caloric intake, diet-induced thermogenesis, thermogenesis, plus the specific nutrients/foods/diets or gut microbiota). No restrictions were placed. Hand-searching the references of the studies and reviews of the field was performed to augment the search strategy. To search for toxicity information (of single foods), the following terms were used: toxicity, adverse events, adverse effects, side effects, reactivity and interactions.

Few papers were available about many topics; therefore, all the research articles were considered with the following scale of priority: systematic reviews and meta-analyses, randomized controlled trials (RCTs), human observational studies, case series, animal studies and in vitro studies.

## 3. The Energy Balance in Humans

In humans, energy intake (EI) and energy expenditure (EE) are in a complex balance, resulting from the difference between EI and EE, aimed at maintaining a relatively constant level of energy stores over time in accordance with the principle of energy conservation [3]. When EI is reduced, a corresponding reduction in EE occurs (and vice versa), in order to minimize perturbations to energy homeostasis [7]. Energy intake is derived from dietary macronutrients (proteins, carbohydrates and lipids) and alcohol. The absorption of calories depends both on food and individual characteristics [3]. A high degree of overfeeding was associated with a greater fractional decrease in stool energy loss in lean but not in obese individuals, thus indicating that the degree of overnutrition relative to individual weight-maintaining energy needs may play a role in the determination of the efficiency of nutrient absorption [8]. Daily total energy expenditure (TEE) can be split into different components: resting energy expenditure (REE), which is the energy required to support body’s basic metabolic activities; activity-induced energy expenditure (AEE), the energy cost of physical activity and exercise; diet-induced thermogenesis (DIT), the energy spent to process the ingested food (about 10%-15% of TEE); and the energy necessary for body thermoregulation. REE can be 3%–10% higher than basal energy expenditure (BEE), which is the energy required to maintain vital body functions [9,10,11]. REE is mostly determined by body size and composition and is positively correlated with body weight and fat-free mass. AEE is the most variable component of TEE depending on an individual’s lifestyle [12,13]. Food intake affects all the components of TEE—but predominantly DIT—with different effects according to the macronutrient composition of a meal and daily variation within the same individual [3,14].

Energy homoeostasis is fundamental for survival and, hence, highly specialized adaptive mechanisms counteract energy imbalances, making energy balance a complex process. Adaptive thermogenesis (AT) and facultative thermogenesis (i.e., the heat production in response to environmental variations) both protect an organism from exposure to cold and regulate the energy balance after dietary changes, and are influenced by the activity of the sympathetic nervous system (SNS), leptin and many hormones (mainly 3,5,3’-tri-iodothyronine) [15,16]. A major site of AT is the brown adipose tissue (BAT), where non-shivering thermogenesis occurs with the uncoupling of mitochondrial substrate oxidation from adenosine triphosphate (ATP) production and the release of fatty acid oxidation energy as heat [17,18]. BAT is activated not only by cold exposure, but also by certain food ingredients, thus contributing to DIT [19,20]. The same signals activating BAT also induce the expression of uncoupling protein 1 (UCP1) in white adipose tissue (WAT) cells (the “beige” cells), a phenomenon known as browning [21].

Either energy restriction or overfeeding induce adaptive changes in the energy balance, with AT, respectively directed towards energy sparing or vice versa. Reducing habitual energy intake by about 10% reduces TEE by 10%–15%, mainly due to reduced REE; furthermore, AT can explain about 50% of the less-than-expected weight loss in patients with obesity [16,22].

## 4. Energy Intake from Food

The actual energy content of some foods may differ from the energy, which is theoretically calculated, due to differences in macronutrient digestibility and food structure [23,24]. One of the best examples of this discrepancy is represented by nuts. Herein, we shortly describe the energy of this paradigmatic food.

Tree nuts are energy-dense foods, due to their high content of lipids (ranging between 40–75 g per 100 g) [25]. However, the inclusion of nuts as part of a healthy diet does not affect body weight, as reported by observational and experimental studies, even though nuts may benefit weight-loss diets [24]. Several mechanisms have been proposed to explain this discrepancy, including appetite control, increased DIT (as discussed below), and discrepancies in available metabolizable energy (ME, i.e., the amount of the food available energy to the human organism) [24]. To calculate the food ME, each energy-contributing food component is multiplied by its Atwater factor [23]. However, recent evidence has demonstrated that the Atwater factors do not provide accurate ME values for several nuts in healthy volunteers [26,27,28,29]. Indeed, based on the measurements on urine and feces, ME values were found to be 25%, 20%, 16% and 5% less than those calculated for almonds, walnuts, cashews and pistachios, respectively. The reason for this discrepancy is partly due to the structure of nuts, which limits the accessibility of digestive enzymes. In oilseeds, such as nuts, lipids are stored in oil bodies which are covered by a thin layer of phospholipids and proteins and encapsulated in cell walls [30], whose components (e.g., cellulose, hemicellulose, peptic substances and lignin) are mainly indigestible by human digestive enzymes [23]. After nut mastication, large particles representing clusters of intact cells remain, which provide protection against disintegration and a physical barrier for enzyme hydrolysis and microbiota metabolism [31]. These clusters of cells, with intracellular lipids encapsulated within the cell walls, were still intact after having passed through the human intestine, thus reducing the intake of energy. Furthermore, it has been demonstrated that when almonds were chewed 10 times, a higher number of larger particles was obtained than when they were chewed 25 or 40 times [32]. These large particles retain more energy and lipids (which are then lost in the stool) than smaller ones (43.7%, 32.7% and 30.8% of the lipid load was lost in the stool after 10, 25 and 40 chews, respectively). On the other hand, other processes (such as roasting) make almonds more brittle and crunchy, with the subsequent production of smaller particles after mastication [33] and the induction of swelling of the cell walls with increased porosity and destruction of oil bodies, favoring the access of digestive enzymes [30]. These changes slightly increased the measured ME of roasted almonds, compared to whole almonds, even though their ME was still lower than that predicted with the Atwater factor (-25% and -19% for whole natural almonds and whole roasted almonds, respectively) [34]. In almond butter, where the cellular structure is fully destroyed, there is a full release of energy, with no discrepancy between the measured ME and the predicted energy content. Similarly, fecal fat content was significantly higher when 70 g of whole peanuts were consumed in healthy adults, compared to other forms of peanuts (i.e., oil, butter and flour) [35].

The effect of structure on the actual energy content of foods has been shown mainly for nuts, but the same effect may be extended to other seeds, legumes and some cereals. This lower actual energy content may have an impact on the overall energy intake when a diet is rich in unprocessed foods where the food structure is retained [23].

## 5. Energy Expenditure Due to Food Intake 

### 5.1. The Role of Nutrients

Food intake stimulates energy expenditure; this is a well-known phenomenon, called DIT or the thermic effect of food. DIT accounts for ~10%–15% of TEE, which is a meaningful amount of the human body daily energy expenditure [36] and which can be measured by indirect calorimetry through the assessment of oxygen consumption and carbon dioxide production [37]. However, this method of measurement based on respiratory exchange has been recently blamed for overestimating DIT, as it is based on the assumption that all metabolic processes of the organism consume oxygen and produce carbon dioxide, which is not always true [38]. Both insulin resistance and, to a lesser extent, abdominal adiposity, have an impact on DIT by reducing the thermic effect of a meal [39]. In fact, insulin, by increasing glucose oxidation and inhibiting lipid oxidation, regulates the cellular substrate flow and utilization, which is therefore impaired in the presence of abnormal insulin sensitivity [40].

At present, there is great interest in the possibility of modulating DIT in order to increase the body's energy expenditure and promote weight loss. First of all, DIT has been proven to be influenced by meal timing, with DIT being higher in the morning and reduced in the evening [41]. Increased nocturnal insulin-resistance and heightened ghrelin levels, slower evening gastric emptying with increased carbohydrate absorption, and increased morning sympathetic activity have been proposed as possible explanations [42]. In addition to meal timing, DIT is influenced by the caloric content of a meal and increases in a direct proportion to the energy intake [14,43]. Finally, the macronutrient composition of food seems to meaningfully affect post-prandial energy expenditure, even if the data in the literature are controversial. Commonly, proteins have been considered to induce an increased energy expenditure which, combined with a higher satiating effect, could determine a higher weight loss [39,44]. On the other hand, carbohydrates and lipids determine a lower DIT than proteins (protein > carbohydrates > lipids) [45,46]. Meals with protein percentages ranging from 11%–30% of the total calories proportionally increase DIT until the value of 30%, where a plateau is reached and a subsequent increase in the protein intake does not increase further the thermic effect of the meal [47]. The protein source should be taken into account, as well: casein, soy or whey proteins are metabolized differently, which may explain the variability in the speed and extent of DIT increase. In particular, whey proteins lead to higher DIT than caseins, while contrasting results have been obtained in the comparison of whey and soya proteins [48]. Regarding the quality of other nutrients, medium-chain lipids seem to heighten DIT more than long-chain triglycerides [49,50] and unsaturated fats more than saturated, probably due to up-regulation of proliferator-activated receptor (PPAR)-α expression [51]. Finally, unrefined, fiber-rich carbohydrates determine an increased energy expenditure, especially if contained in low-processed foods [39].

Overall, very few data are available about this topic and the clinical significance of any single nutrient or single meal is unclear in a weight-loss strategy.

### 5.2. The Role of Foods

**Figure 1:**
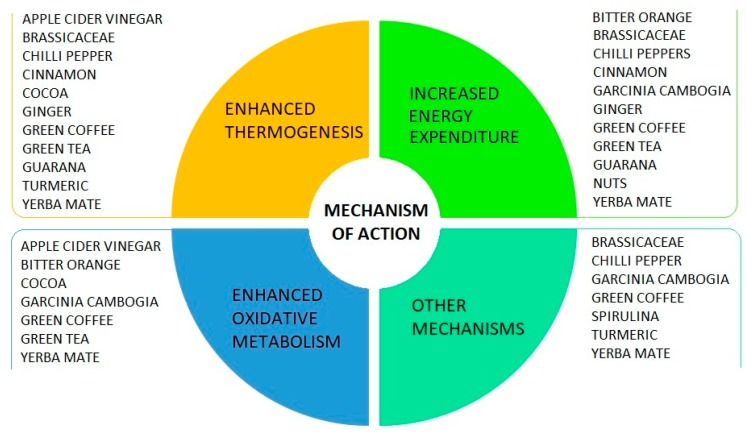
Supposed mechanisms of action of food impacting on energy balance.

An increasing number of foods are supposed to increase human energy expenditure [52]. The list is long and is gradually getting longer (Supplementary Table S1). Herein, we examined those for which scientific studies were available (Figure 1).

#### 5.2.1. Green Coffee

##### Available Evidence

Almost the whole world’s coffee consumption derives from the beans of two coffee plants—*Coffea canephora* and *Coffea arabica*—which contain many bioactive compounds, such as caffeine (1,3,7-trimethylxanthine) and chlorogenic acid [53]. Green (unroasted) coffee and roasted coffee contain the same amount of caffeine (1.2%–2.2%) but a different percentage of chlorogenic acids (6.5%–10% vs 2.7%–3.1%, respectively) [54]. Caffeine increases thermogenesis and energy expenditure by several mechanisms [55,56]. In humans, the thermic effect lasts about 150 min after a single-dose caffeine ingestion [56] and one RCT reported a stronger metabolic impact among habitual low consumers of caffeine, thus suggesting the possibility of a long-term insensitivity to the effects of caffeine after high and prolonged exposure [57]. In human trials, an increase in energy expenditure has been reported, varying from 6% (after 50 mg caffeine intake) [58] to 7% (after 200 mg caffeine consumption) [57]. Chlorogenic acid has been reported to have beneficial effects against obesity and other dysmetabolic disorders, as well as playing a favorable role in energetic metabolism in both human and animal studies [59,60]. In a pilot study, the consumption of 1 cup of green coffee (containing 6 mg caffeine per kg of lean body mass, about 215–280 mg) determined an increase of REE by 6.4% at 30 min and 2.2% at 180 min, with a positive correlation between the chlorogenic acid assumed and the REE values at 30 min [61].

##### Molecular Mechanisms of Action

In cultured adipocytes, caffeine has been shown to enhance BAT function and thermogenesis by up-regulating UCP1 and BAT-selective regulatory genes including PPAR-γ, PPAR-γ coactivator (PGC)-1α and PR domain containing 16 (PRDM16) [55]. PGC-1α also induces mitochondrial biogenesis and stimulates fatty-acid oxidation and oxygen consumption through the co-activation of PPAR-γ [55]. Caffeine induces PGC1α and UCP1 indirectly as well, by antagonizing the transient receptor potential vanilloid (TRPV)-4, a negative regulator of PGC-1α in the TRPV receptor family and a modulator of beige/brown adipocyte thermogenesis [55]. Adipose tissue browning is additionally stimulated through the expression of other specific genes (*CD137*, *LHX8*, *P2RX5*, *CITED1* and *COX8b*) [55]. Further mechanisms have been implicated in the caffeine-induced thermogenesis, such as the antagonism of adenosine-mediated inhibition of the secretion of epinephrine and norepinephrine and the inhibition of phosphodiesterase, which increases intracellular levels of cyclic adenosine monophosphate (cAMP). Catecholamines stimulate β-adrenergic receptors and cAMP activates the protein kinase A, which enhances UCP1 activity through increased free fatty acid release [55,58,62]. Chlorogenic acid principally up-regulates the AMP activated protein kinase (AMPK) with increased fatty acid oxidation and ATP production [63].

##### Toxicity and Reactivity

According to the Food and Drug Administration (FDA), European Food Safety Authority (EFSA) and Health Canada, the consumption of up to 400 mg caffeine can be considered safe in healthy adults, without overt, adverse cardiovascular, behavioral, reproductive, bone and developmental effects [64,65,66]. No reactivity with drugs, supplements or food has been reported for green coffee.

#### 5.2.2. Green Tea (*Camellia sinensis*)

##### Available Evidence

The leaves of the plant *Camellia sinensis* give the three most popular types of tea, green (unfermented), black (fully fermented) and oolong (semifermented) [67]. The main components of green tea are polyphenols, in particular flavon-3-ols, also known as catechins, mostly epigallocatechin-3-gallate (EGCG) [68]. Caffeine is naturally contained in green tea as well, in a variable amount, according to the brewing period and the tea and water rate [68]. The fermentation process lowers the content of polyphenols and increases caffeine in tea; green tea contains two times more catechins, but 2–3 times less caffeine than black tea [68,69]. Indeed, most of the studies available on the effects of tea on the energy balance are related to green tea. Many *in vitro* and animal studies have documented enhanced thermogenesis, heightened energy expenditure and fat oxidation after green tea consumption [68,70,71]. Other beneficial effects are a reduction in fat mass due to the interruption of lipid emulsification, the inhibition of gastrointestinal digestive enzymes activity, improvements in the gut microbiota and reduction in adipocyte differentiation and food intake [69,71,72]. Both human observational studies and trials have confirmed increased energy expenditure after the acute administration of green tea, but the long-term effects are not currently proven [69,73,74,75,76]. It is noteworthy that in most of the trials, the volunteers were provided with high doses of green tea catechins, equivalent to 3–4 cups of brewed green tea a day [72], which are usually consumed only by a few population groups.

##### Molecular Mechanisms of Action

Catechins and caffeine (see the previous paragraph) affect energy expenditure differently. Catechins inhibit the catechol-O-methyl transferase enzyme (COMT) in almost all the tissues, which, in turn, inhibits the degradation of norepinephrine and produces protracted β-adrenergic stimulation. Hence, the SNS activity is increased along with energy expenditure and fat oxidation [68,72,73]. In vivo and in vitro, EGCG has been shown to affect energy expenditure by the activation of AMPK, which promotes fatty acid oxidation and ATP production [69,72]. EGCG has also been reported to inhibit mitochondrial oxidative phosphorylation, up-regulate the gene expressions of UCPs in BAT and decrease ATP levels, which activates AMPK [69,72,74]. In vitro, fermented green tea induced the up-regulation of fatty acid oxidation-related genes and increased energy expenditure by inducing serotonin secretion [70].

##### Toxicity and Reactivity

Green tea is safe across a wide range of intakes and preparations, however concentrated solid extracts are less tolerated due to the high content of EGCG [77]. Gastrointestinal symptoms, such as nausea/vomiting, diarrhea, flatulence, abdominal bloating and dyspepsia have been reported after the intake of high doses of beverages or extracts (corresponding to 5–6 L of beverage/day) [76]. The intake of 10–29 mg/kg/day of green tea-based dietary supplements has resulted in liver toxicity due to oxidative stress and cytotoxic damage [67]. The caffeine content in green tea is low but, depending on self-sensitiveness to methylxanthines and doses, symptoms such as nervousness, restlessness, tremors, palpitations, sleep disorders, vomiting, diarrhea, headaches, epigastric pain and tachycardia have also been reported [67]. In adults an intake of ~300 mg EGCG/day in solid bolus dose and ~700 mg for tea preparations are considered safe [77]. The vitamin K contained in green tea leaves can antagonize the effect of anticoagulants [67].

#### 5.2.3. Cocoa and Dark Chocolate

##### Available Evidence

Cocoa, the main constituent of dark chocolate derives from the *Theobroma cacao* tree. Dark chocolate is considered a functional food, due to its content of fatty acids, vitamins, minerals, fiber, several methylxanthine alkaloids (4% of the dry weight), mainly caffeine and theobromine [78,79] and polyphenols (12%–18% of dry weight); in particular, flavan-3-ols, (+)-catechin and (−)-epicatechin and B-type procyanidins [80,81,82]. Cocoa has been reported to reduce fatty acid synthesis and transport systems, enhance β-cell function, down-regulate insulin receptor kinase activity, improve peripheral insulin sensitivity, inhibit digestive enzymes and increase thermogenesis in liver and WAT both in animals and humans [83,84,85,86]. A meta-analysis of human RCTs reported that cocoa/dark chocolate supplementation do not affect anthropometric measures in adults; however, a subgroup analysis indicated that ≥30 g dark chocolate per day for at least 4 weeks had favorable effects on weight and body mass index (BMI) [84]. Studies in mice have reported that procyanidins of cocoa liquor (the pure cocoa mass derived from cocoa beans), in addition to thermogenic effects, have a role in the prevention of postprandial hyperglycemia by increasing glucagon-like peptide-1 activity, phosphorylation of the AMPKα and glucose transporter type-4 translocation in skeletal muscle and BAT [87].

##### Molecular Mechanisms of Action

Cultured WAT cells of cocoa-fed rats have shown the upregulation of the gene expression of UCP2, a homolog of UCP1 implicated in non-shivering thermogenesis [83]. Procyanidins affect energy expenditure by inducing the gene and protein expression of UCPs (UCP1, UCP2 and UCP3), AMPKα and PGC-1α in adipose, liver and muscle tissues [88]. Once activated, AMPK leads to the inhibition of energy-consuming biosynthetic pathways, such as fatty acid and sterol synthesis and the activation of ATP-producing catabolic pathways, such as fatty acid oxidation [89]. PGC-1α increases mitochondrial biogenesis and the expression of UCPs, promoting fatty acid oxidation as well [88]. Furthermore, the methylxanthines contained in dark chocolate act as adenosine receptor blockers in vivo [90], affecting energy expenditure by stimulating basal and noradrenaline-stimulated lipolysis in rat fat cells [91]. Xanthine derivatives induce secretion of catecholamines, which bind to adipose cells and increase thermogenesis by increasing the expression of thermogenic genes and releasing free-fatty acids which, in turn, enhances UCPs [92]. Studies in humans are needed to confirm these mechanisms.

##### Toxicity and Reactivity

Depending on the percentage of dry cocoa, chocolate may contain trace heavy metals–principally cadmium and lead–resulting from the contamination of the soil or during manufacturing processes [93]. European Legislation has set the levels to 1 mg/kg for cadmium and 0.3 mg/kg for lead as the maximum tolerable amount in cocoa powder [94].

#### 5.2.4. Yerba Mate (*Ilex paraguariensis*)

##### Available Evidence

The infusion (mate) derived from the dried leaves of Yerba mate is widely consumed throughout South America as well as in many other countries. Its numerous beneficial effects are likely due to the content of several bioactive compounds, such as polyphenols, alkaloids, soaps, triterpenoids, flavonoids and chlorogenic acid [95,96]. In one placebo-controlled study, an infusion containing 1.5 g mate dry extract increased REE by almost 5% and resulted in a 5% reduction of the respiratory quotient in non-obese women and men, probably through increased lipid oxidation capacity [97]. In humans, pharmacological doses of Yerba mate extracts acutely induced a significant increase in the exercise energy expenditure due to the preferential use of fatty acids as an energy substrate [98]. Chronically, these extracts determined an increase in REE, thermogenesis in WAT and a reduction in the WAT synthesis of fatty acids in mice, leading to weight and fat loss and lower circulating leptin levels [99].

##### Molecular Mechanisms of Action

Chlorogenic acid, as already reported for green coffee, increases fatty acid oxidation by upregulation of AMPK [63] and inhibits adipogenesis by down-regulation of the expression of specific genes, such as *Creb-1* and *C/EBPa* [100]. The increased thermogenic effects after supplementation with Yerba mate extracts seem to be due to increased mitochondrial genesis and expression of UCPs, resulting in greater efficiency in the mitochondrial respiratory chain and heat dissipation in mice fed with high-fat diet [101].

##### Toxicity and Reactivity

The metabolic effects of Yerba mate have been obtained by using supplements with high doses of the active compounds, thus not well representing the effect of the natural food for which human data are still lacking. Toxicological investigations in rats have reported a good tolerability of single (up to 2 mg/kg dose) and chronic administration of Yerba mate extracts, which seem to be safe for consumption at dosages up to 300 mg/kg/day in pregnant rats [102,103].

#### 5.2.5. Bitter Orange (*Citrus aurantium*)

##### Available Evidence

*Citrus aurantium*, better known as bitter orange, is an evergreen plant whose fruits have been used for many centuries both as a food in Southern Europe and as a supplement in traditional medicine in China and South America [104,105]. These fruits contain alkaloids -particularly synephrine and octopamine—and other compounds, such as flavonoids -in particular hesperidin, naringin, limonene and tangaretin—with potential beneficial effects on metabolism and health [106,107]. A few human studies have demonstrated both an acute thermogenic effect with a statistically significant increase in REE, DIT and blood catecholamines levels, as well as weight loss and appetite suppression after the ingestion of bitter orange extracts [104,107,108,109,110]. However, long-term data are lacking, as well as data about the effects of the consumption of the fruit by itself, as the available studies have employed dry and purified extracts from the orange peel, containing a high dose (~26 mg) of p-synephrine.

##### Molecular Mechanisms of Action

Synephrine and octopamine, are contemporary α- and β-adrenergic agonists which display sympathomimetic effects by contributing to oxidative metabolism, lipolysis promotion and β3- and α-adrenergic receptor stimulation [106]. The anti-adipogenic effects of *p*-synephrine in 3T3-L1 preadipocytes are due to the regulation of the Akt signaling pathway and the suppression of adipogenesis-related proteins [111]. After treatment with *Citrus aurantium*, primary cultured brown adipocytes displayed increased differentiation associated with the elevation of thermogenic factors including UCP1 and PPAR coactivator 1α, by AMPK activation [112].

##### Toxicity and Reactivity

Case reports, as well as animal and human studies, have provided evidence for cardiovascular effects due to the ingestion of high synephrine doses contained in supplements, especially in combination with caffeine [106,109,113]. The dietary exposure occurring through ingestion of the citrus fruits is much lower, with the median total daily intake of synephrine being up to 6.7 mg/day, and the safety issues are less evident [109,113].

#### 5.2.6. Ginger

##### Available Evidence

Ginger (*Zingiber officinale*) is a plant from the Zingiberaceae family, native to South Eastern Asia, which is widely used for food, flavoring and as a medicine in China and India historically [114]. A few small cross-over human trials have studied the effects of ginger on energy expenditure [115,116], with contrasting results, differently from animal [117,118,119,120,121,122] or in vitro studies [123], which showed improved energy expenditure, lower weight gain, increased browning of WAT and promotion of mitochondrial biogenesis. The contrasting human data, showing either an increased DIT [87] or no thermogenic effects [116], do not allow us to obtain definitive conclusions.

##### Molecular Mechanisms of Action

Ginger enhances thermogenesis, increased mitochondrial biogenesis, enhanced BAT function and activated WAT browning in animals through the activation of the sirtuin-1 (SIRT1)/AMPK/PGC-1α pathways [118,121,122]. The mRNA expression of Sterol regulatory element-binding protein 1 (SREBP-1c) in the liver and leptin in adipose tissues were downregulated, while those of adiponectin, hepatic carnitine palmitoyltransferase1 (CPT-1), acyl-coA oxidase (ACO), Glucose transporter 2 (GLUT-2) and pyruvate kinase (PK) were upregulated after ginger treatment in rats, thus supporting an effect of this compound at the transcriptional level of energy metabolizing proteins [119]. An increase in cellular fatty acid catabolism via the activation of the PPARδ pathway has been shown in mice treated with ginger extracts [120].

##### Toxicity and Reactivity

Apart from characteristic burning sensation felt upon the consumption of ginger [115], no adverse effects or toxicity has been reported in the human studies. A recent systematic review has also shown its safety in pregnancy [124].

#### 5.2.7. Curcuma Longa

##### Available Evidence

Turmeric (*Curcuma longa*) is an herbaceous plant of the ginger family (*Zingiberaceae*) that has been used both as a flavoring and a stimulating agent [125]. Curcumin, also known as diferuloylmethane, is a natural flavonoid component of turmeric, whose antioxidant, anti-inflammatory, antibacterial, anticancer, insulin-sensitizing and hypoglycemic properties have been demonstrated in many studies [126,127,128]. One animal [129] and one in vitro [130] study showed that curcumin promotes the browning of WAT, while one observational human study [131] has reported that the supplementation of an extract of *Curcuma* reduced the urinary excretion of niacin metabolites and medium- and short-chain acylcarnitines; thus suggesting the potential induction of mitochondrial β-oxidation of fatty acids for energy production. Therefore, the evidence relative to a thermogenic role of curcumin is still scarce.

##### Molecular Mechanisms of Action

The following mechanisms have reported for curcumin: increase of thermogenic gene expression, enhanced mitochondrial biogenesis, promotion of the expression of β3-adrenoreceptors with increased levels of plasma norepinephrine [129], increased levels of hormone-sensitive lipase and p-acyl-CoA carboxylase with enhanced lipolysis, increased expression of UCP1 by AMPK activation [130] and upregulation of the cAMP/protein kinase A (PKA)/cAMP response element-binding protein (CREB) pathway, which plays an important role in energy expenditure and thermogenesis [132].

##### Toxicity and Reactivity

Turmeric has been reported to contain many toxic, mutagenic, carcinogenic and hepatotoxic components [133]. Overall, human studies have reported mild adverse effects after curcumin supplementation, with gastrointestinal upsets being most common [134]. The long-term consumption of high doses of curcumin may be dangerous and case reports of acute liver injury have been described [133]. Owing to its inhibitory effect on cytochromes P450, turmeric can potentially interact with many drugs, such as anticoagulants, antibiotics, cardiovascular drugs, anticancer drugs and antidepressants and interactions with clopidogrel, warfarin and etoricoxib have been reported [134,135].

#### 5.2.8. Cinnamon

##### Available Evidence

Cinnamaldehyde is a compound found in cinnamon responsible for its particular flavor, which may improve metabolism owing to its reported hypoglycemic and lipid-lowering effects [136]. Two small randomized human clinical trials in healthy subjects showed that the acute ingestion of extracts of cinnamon (cinnamaldehyde [137] or cinnamyl isobutyrate, respectively [138]) increased energy expenditure (evaluated by indirect calorimetry) by ~3.6 kcal over 90 min from ingestion [137] or reduced short-term energy intake by 4.6% [138], when compared to placebo. These changes are too small to be clinically relevant. Animal studies have demonstrated that extracts of cinnamon elicit thermogenesis responses [139], reduced visceral adiposity, attenuated hyperphagia and normalized energy efficiency [140] and attenuated obesity through the modulation of genes implicated in the lipid metabolism pathways [141]. Currently, chronic studies conducted with the cinnamon amount usually consumed in an everyday diet are lacking.

##### Molecular Mechanisms of Action

In rats, cinnamon-linked increased rate of cold adaptive thermogenesis was due to the elevation in norepinephrine, blood levels of free fatty acid levels and increased expression of UCP1 in BAT [142]. Experimental studies have reported the ability of cinnamaldehyde in activating phospho-AMPK in adipose tissue [140], enhancing thermogenic and metabolic responses in human subcutaneous fat cells through a cAMP dependent protein kinase/p38 mitogen-activated protein kinase (p38 MAPK)-dependent pathway (involved in the transcription of thermogenic genes) [143] and inducing browning in mice subcutaneous adipocytes by increased expressions of UCP1 and other brown adipocyte markers and involvement of the β3-adrenoreceptor activity [144]. Finally, cinnamaldehyde has been shown to activate the transient receptor potential ankyrin 1 (TRPA1), an ion channel located at the cellular surface, acting as a mechanical and chemical stress sensor, which is involved in adrenalin secretion [145].

##### Toxicity and Reactivity

Cinnamon is obtained from different tree species of the genus *Cinnamomum*: Chinese cinnamon (*Cinnamomum cassia* or *Cinnamomum aromaticum*), coming from the East and containing high level of coumarin, with potential harmful effects [146]; and Ceylon cinnamon (*Cinnamomum zeylanicum* or *Cinnamomum verum*), coming from Sri Lanka and Madagascar, which contains only trace amounts of coumarin. Hepatotoxicity, effects of coumarin on coagulation and potential interference with drugs and mild adverse events have been reported for Chinese cinnamon, while, the consumption of Ceylon cinnamon seems safe [147,148,149].

#### 5.2.9. Chili Pepper (Capsicum Species)

##### Available Evidence

Chili peppers are common food flavoring, which are also used as a traditional medicine in some cultures [150,151]. Chilis contain pungent capsaicinoids (capsaicin and dihydro-capsaicin), the major bioactive compounds responsible for the hot taste sensation, non-pungent capsaicin analogs, named capsinoids (e.g., capsiate, dihydro-capsiate and nordihydro-capsiate); and antioxidants, vitamins and carotenoids [150]. Studies in humans investigating a wide range of chili doses have shown that the weight-loss properties of chili are due to enhanced energy expenditure and thermogenesis [152,153]. Conflicting results have been found on the properties of capsaicin and capsiate in decreasing the respiratory quotient by enhancing fat oxidation, due to the different designs of the studies, the body composition and BMI of the subjects included and the habitual consumption of chili in their diet [152,153,154]. Interestingly, the consumption of 2.56 mg of capsaicin (1.03 g of dried red chili pepper) per meal was able to mitigate the unfavorable negative energy balance effect of decrease in DIT and REE induced by a 25% caloric restriction in humans [155]. However, the doses required to impact metabolism is high and out of the tolerated range for most people [137].

##### Molecular Mechanisms of Action

In mice, dietary capsaicin activated thermogenesis in WAT by up-regulating the expression of SIRT1 and PGC-1α, both of which increase the expression of UCP1 and bone morphogenetic protein-8b, resulting in energy dissipation by thermogenesis, increased EE and metabolic activity [156]. Both in mice and humans, capsaicin and capsinoids enhance energy expenditure by triggering BAT through multiple mechanisms, such as the stimulation of non-shivering thermogenesis by binding to TRPV1, stimulation of the SNS and catecholamine secretion from the adrenal gland [19,137,152,153,154,156,157].

##### Toxicity and Reactivity

In humans, one milligram of capsaicin has neither adverse effects nor affects energy expenditure [137]. Side effects with higher doses of capsaicin, even when provided in capsule form (up to 135 mg/day) include low palatability, gastric distress, dyspepsia, anal burning, bowel irregularities and diarrhea [153]. Capsiate is more tolerable due to its non-pungent characteristics deriving from rapid hydrolysis in the oral cavity, with reduced accessibility to nociceptors [153,154].

#### 5.2.10. Garcinia cambogia

##### Available Evidence

*Garcinia cambogia* is an herbal product derived from the fruit of the Malabar tamarind tree (also called *Garcinia gummi-gutta*) native to India, Nepal and Sri Lanka [158]. The fruit rind is used either as food preservative, flavoring agent, food-bulking agent or traditional medicine in many Asian countries [159]. *Garcinia* contains xanthones, benzophenones, amino acids and organic acids, of which hydroxy-citric acid (HCA) accounts for 10%–30% of the weight of *Garcinia* fruit and 20%–60% of the extract [158].

Studies with different duration of administration and doses of *Garcinia cambogia* or its extract, were performed both in animals and humans with conflicting results. Favorable effects of *Garcinia cambogia* on glucose and lipid metabolism, as well as on appetite reduction, have been reported [159,160,161].

However, no beneficial effect on EE has been found at different doses and durations of HCA supplementation in human trials, both in the short period and up to 12 weeks [162,163,164]. A recent meta-analysis of human trials failed to find a significant weight-loss effect of supplementation with *Garcinia cambogia* [165].

##### Molecular Mechanisms of Action

In animal studies, supplementation with HCA induced energy expenditure acceleration by the activation of the adiponectin AMPK signaling pathway [166] or through the regulation of thyroid hormone levels [167]. HCA inhibits serotonin uptake leading to satiety and reduced food intake and down-regulates ATP-citrate lyase, increasing fat oxidation and decreasing *de novo* lipogenesis [159,160,161].

##### Toxicity and Reactivity

Supplements containing a standardized dose (e.g., 300–500 mg) of *Garcinia cambogia*-derived HCA, consumed up to three times daily, are considered safe [160]. In one human trial, HCA has been supplemented with doses up to 5600 mg/day without adverse effects [168]. However, case reports have described severe hepatotoxicity, including acute liver failure requiring liver transplantation and acute necrotizing eosinophilic myocarditis in subjects using pure *Garcinia cambogia* supplements [169,170,171,172]. A natural product for weight loss containing *Garcinia cambogia* and a variety of other ingredients has been associated to fatigue, nausea, vomiting, colic, fever, chills, anorexia, abdominal pain, jaundice, increased levels of liver enzymes and bilirubin in healthy adults [173]. Liver toxicity has been associated with both cholestatic and hepatocellular patterns of injury.

#### 5.2.11. Guarana (*Paullinia cupana*)

##### Available Evidence

Guarana is a plant native to the Amazon basin, which is largely used by beverage industries [174]. Guarana seeds contain the highest percentage (2%–8%) of caffeine, compared to any other plant, a high concentration of polyphenols (particularly proanthocyanidins) and small quantities of other stimulant purine alkaloids, such as theobromine and theophylline [174,175]. Two human RCTs reported increased energy expenditure [176] and short-term weight and fat loss [177] after Guarana extract administration. In one animal study, Guarana seed powder supplementation prevented weight gain, insulin resistance and adipokine dysregulation induced by a Western diet [178]. However, conclusive results regarding Guarana supplementation on weight management are still lacking.

##### Molecular Mechanisms of Action

Guarana exerts an anti-adipogenic activity by down-regulating the expression of pro-adipogenic genes, up-regulating the expression of anti-adipogenic genes and increasing β-catenin nuclear translocation, which may contribute to adipogenesis inhibition [179]. Guarana induced BAT expansion, mitochondrial biogenesis, UCP1 overexpression, AMPK activation and minor changes in gut microbiota in rats [178]. Metabolic effects after Guarana supplementation are mainly due to its high caffeine content.

##### Toxicity and Reactivity

The European Medication Agency (EMA) recommends a maximum intake of 2250 mg/day of Guarana extract, due to its high percentage of caffeine, which is considered safe up to a dose of 400 mg/day [64,65,66]. Supplements containing guarana, together with multiple ingredients—above all, high doses of caffeine—have determined agitation, anxiety, insomnia, aggressivity, decreased blood bicarbonate and tachycardia up to cardiorespiratory arrest [66,180]. In rats, pharmacological interactions have been reported after administration of guarana supplements, either with central nervous system stimulants or lamotrigine and amiodarone, leading to exacerbation of seizures and risk of arrythmias [66].

#### 5.2.12. Brassicaceae

##### Available Evidence

Broccoli is a vegetable of the *Brassicaceae* (or *Cruciferae*) family, which contain sulfur-based compounds named glucosinolates [181]. These compounds are hydrolyzed to biologically active isothiocyanates (ITC) by the action of myrosinase, a vegetable enzyme present in the human gut microbiota [182,183]. Glucoraphanin, the predominant glucosinolate in broccoli, releases the ITC sulforaphane (SFN) [184]. A 100-g serving of fresh broccoli can release 37–75 mg of SFN, but a therapeutic dose of SFN may not be achieved by a regular diet, as transportation, storage conditions, preparations and cooking, may decrease the vegetable content of SFN [182]. In vitro and animal studies have reported a beneficial effect of SFN on lipid metabolism and thermogenesis [185,186,187,188], as well as on gut microbiota [189]. However, extrapolating these results to humans is difficult because studies are still lacking.

##### Molecular Mechanisms of Action

In vitro studies have shown that SFN can induce apoptosis in adipocytes [185], inhibit adipocyte differentiation and promote lipolysis in adipocytes [186]. Both glucoraphanin and SFN exert thermogenic effects. SFN increased both mitochondrial biogenesis and function by up-regulating nuclear factor erythroid 2 (NF-E2)-related factor 2 (Nrf2) /SIRT1/ PGC-1α signaling [187], as well as enhancing UCP1 expression through the activation of the Nrf2; thus promoting browning of WAT [188].

##### Toxicity and Reactivity

Brassicaceae are considered a goitrogenic food due to the ability of the goitrin, a molecule derived from myrosinase hydrolysis of the glucosinolates progoitrin, to inhibit iodine utilization by the thyroid [190]. It is worth noting that the effects of overactivation of the Nrf2-related metabolic pathways are controversial, as the worsening of insulin resistance, as well as glucose and lipid metabolism, have been reported in mice [191].

#### 5.2.13. Nuts

Nut-rich diets have been proved to provide positive effects, both on cardiovascular health (owing to their content of mono- and polyunsaturated fats, flavonoids and vitamins) and on body weight, BMI or waist circumference [24,192]. At present, the role of nuts in the regulation of energy balance has not been extensively studied; however, favorable effects have been reported by a couple of RCTs [51,193]. In particular, a 28% increase in DIT 5 h after a meal rich in walnuts and a ~100 kcal higher BEE after 2 months of a nut-rich diet (independent of weight change) have been observed [193]. The role of peanuts is even more uncertain as, in a small RCT, the consumption of high-oleic peanuts increased DIT more than conventional peanuts, but similarly to controls consuming biscuits [194].

#### 5.2.14. Apple Cider Vinegar

Apple cider vinegar is a rich source of polyphenols and acetic acid [195]. A systematic review and metanalysis of human trials failed to reach conclusive results on short and long-term blood glucose control after the administration of a wide range of dosages of apple cider vinegar [196]. At present, studies about the effects of apple cider vinegar on human energy expenditure are lacking. In Wistar rats subjected to a high-fat diet, the supplementation of apple cider vinegar (7 mL/kg/day) for 30 days reduced BMI, abdominal circumference and improved satiety [197]. In vitro, acetic acid upregulates the expression of genes for fatty acid oxidation enzymes and thermogenic proteins (e.g., ACO, CPT-1, and UCP2 through α2-AMPK/PPARα-mediated pathway [198]. The lack of human studies on the role of apple cider vinegar on energy balance makes it impossible to draw conclusions about the potential effects of this food.

#### 5.2.15. Spirulina

Spirulina refers to a large number of photosynthetic eubacterial species belonging to the phylum Cyanobacteria (*Arthrospira platensis* and A. *maxima*) [158]. These microscopic blue-green algae are a source of high-quality proteins, and contain nearly all essential amino acids, vitamins, minerals, fiber and bioactive compounds [199]. A metanalysis of five human trials (278 subjects) found that the chronic administration of spirulina at variable doses (from one to 4.5 g/day) significantly reduced body weight, body fat percentage and waist circumference [200]. Interestingly, the weight loss was not dose-dependent and was higher in patients with obesity rather than in those with overweight [201]. Many beneficial effects, both on glucose and lipid metabolism and oxidant status, have been described in human studies [200,201,202]. However, no data about the potential role of spirulina on energy expenditure are available; therefore, at present, no compelling evidence on this topic is available.

#### 5.2.16. Foods without Scientific Evidence to Date

A long list of foods that have been proposed as “fat-burning” or “slimming” agents have not been the subject of scientific studies supporting these supposed benefits. Among these are pineapple, bitter pumpkin (*Momordica charantia*), mangosteen, *Griffonia simplicifolia*, *Rhodiola rosea*, *Hoodia gordonii*, *Fucus vesciculosus*, *Cissus quadrangularis*, *Irvingia gabonensis*, *yohimbine*, *Caralluma fimbriata*, *Coleus forskohlii* and avocado (*Persea americana*). These foods do have other potential beneficial properties for human health but, to date, their effectiveness in inducing weight loss is far from proven.

### 5.3. The Role of Diet Plans

A few human trials have compared the individual energy expenditure under different dietary regimens. A systematic review and meta-analysis of 32 controlled-feeding studies with 563 participants found no effects on TEE of low-carb versus low-fat diets with equivalent protein content [203]. A few RCTs found a significantly lower TEE decrease with low-carb diets when compared either to high-carb [204] or low-fat [205] diets. However, the pooled weighted mean difference in energy expenditure reported in the metanalysis was negligible (26 kcal/day) and favoring low-fat diets [203].

More recently, Ebbeling et al. measured TEE with doubly labeled water in 162 overweight/obese adults randomized to three diets with similar protein and energy content but different carbohydrate percentage (high, 60%; moderate, 40%; and low, 20%) [206]. TEE was increased by 52 kcal/day for every 10% decrease in the proportion of carbohydrates from the diet [206]. Participants following the low-carb diets showed significantly lower circulating leptin and ghrelin levels, affecting both hunger and energy expenditure [207,208]. This study supported the so-called “carbohydrate-insulin model”, according to which a reduced proportion of dietary carbohydrate drives lower insulin secretion and increases fat mobilization and oxidation, thus leading to enhanced energy expenditure [209,210,211]. However, this trial has been criticized due to deviations from the planned analyses, the inclusion of subjects with excessive unaccounted energy and other methodological problems [212].

Therefore, more extensive and methodologically rigorous trials are needed before definitive conclusions on this topic can be reached. At present, the recommendation of combining a proven healthy diet with a daily exercise to obtain/maintain an adequate body muscle mass remains the best method to prevent a decline in energy expenditure after weight loss.

## 6. The Impact of the Gut Microbiota on the Human Energy Balance

The gut microbiota, which is the microbial community populating the digestive tract, regulates both metabolism and energy balance in a symbiotic relationship with the human host. Micro-organisms extract energy from foods that humans cannot digest, producing bioactive compounds such as short-chain fatty acids (SCFAs)—mainly acetate, propionate and butyrate—which supply energy to the intestinal epithelium and liver, providing ~10% of the daily caloric requirement [213]. The microbiota regulates energy balance through different mechanisms: gut–brain axis control at both the level of intestinal nutrient-sensing mechanisms, as well as at the central nervous system integration sites; development of a low-grade chronic systemic and adipose inflammation together with abnormal gut permeability by an increased relative abundance of pathogenic bacteria; effects on the metabolism of bile acids, with the production of secondary bile acids activating thyroid hormones and oxygen consumption; and impaired secretion of gut peptides and hormones implicated in appetite regulation [214,215,216]. Alterations in gut–brain signaling can affect the regulation of food intake and SCFAs impact on the incretins and hormones implicated in energy homeostasis, such as glucagon-like-peptide 1, gastric inhibitory peptide, peptide YY, leptin and insulin [217]. The gut microbiota of obese mice has been shown to display a higher capacity to harvest energy from the diet and genes related to phosphotransferase systems involved in microbial carbohydrate processing have been found to be increased in both obese mice and humans [215]. Indeed, the relevance of these processes in the energy balance control has been discussed [218] and an increased pro-inflammatory microbiota, together with the impaired secretion of gut peptides and hormones, seem to be the main mechanisms linking dysbiosis to the occurrence of dysmetabolic diseases [215].

Data relative to the role of microbiota in energy expenditure are controversial. The SCFA turnover has been estimated to account for approximately 7% of REE [219], while gut microbiota composition was not associated with REE level [220].

Supplementation with butyrate enhanced energy expenditure in mice by induction of mitochondrial function in brown fat and skeletal muscle, with increased thermogenesis and fatty acid oxidation [221]. Supplementation with acetate, the most abundant SCFA in the colon (accounting for more than half of the total fecal SCFAs), induced browning by altering the expression of genes involved in beige adipogenesis [222]. An altered nutrient load induced rapid changes in the human gut microbiota composition, these changes being directly associated with stool energy loss in lean individuals, such that a 20% increase in Firmicutes and a corresponding decrease in Bacteroidetes was associated with an increased energy harvest of ≈150 kcal [8]. The bacterial endotoxin lipopolysaccharide—produced by the large gut community of Gram-negative bacteria—binds and activates Toll-like receptor 4, leading both to the repression of adaptive thermogenesis through endoplasmic reticulum stress-mediated mitochondrial dysfunction [223], and the suppression of white adipose tissue browning [224]. Intriguingly, obesity-induced alterations of the gut microbiome persist after successful dieting in obese mice and contribute to weight regain, as persistent dysbiosis contributes to diminishing post-dieting flavonoid levels and reducing energy expenditure [225].

Recently, a stratification of individuals in enterotypes by gut microbiota composition has been proposed, with the most important patterns being the P-type (dominated by Prevotella) and the B-type (dominated by Bacteroides), which probably exist as a continuum, rather than separate entities [226]. The P-type—characterized by hydrolase activity—has been associated with a high-fiber and resistant starch rich diet; while the B-type—characterized by saccharolytic and proteolytic capacity—has been associated with high-fat, low-fiber, Western-type diets [226]. In response to arabinoxylans from grain bran, P-type individuals produced larger amount of SCFAs (especially propionate) and showed higher weight loss and improvements in glucose metabolism when compared to B-type individuals [227,228,229]. On the other hand, B-type individuals lose more weight on a bifidogenic diet (rich in inulin and oligosaccharides) [226]. Should these data be confirmed in larger samples, they suggest a differential individual response to the same food, according to gut microbiota composition and ability to metabolize food, extracting more or less energy from it. This is in line with emerging concepts of a need for personalized nutrition [230]. Furthermore, complexity in individual microbiota introduces variability and errors in the measurement of energy expenditure, which usually are not considered and controlled for, making prediction of the effects of nutrition on the human energy balance extremely complex [11].

## 7. Conclusions

In Western societies, the availability of highly processed food and general lifestyle have concurred to generate an obesity pandemic. In attempts to address unavoidable weight gain, the general population has been fascinated by foods that can increase energy expenditure. However, only a few foods can potentially affect energy expenditure—usually when consumed in much higher amounts than those usually consumed. In humans, energy balance is complex and multifactorial and physiological compensation occurs with changes in energy intake and/or expenditure. Moreover, other factors such as microbiota composition and activity are involved, influencing food metabolism and nutrient utilization. Any attempts to classify diets and foods based on supposed roles in energy balance implies an excessive simplification of real biologic complexity, which we are just beginning to understand. Long-term and well-designed human intervention trials in different population groups are important to draw any conclusions on the effect of foods and dietary regimens in energy balance.

## Supplementary Materials

The following are available online at https://www.mdpi.com/2072-6643/12/4/1161/s1, Table S1: Effects on energy expenditure and hypothesized mechanisms to induce weight loss.
