# Supplementary material for: A Critical Review on the Role of Food and Nutrition in the Energy Balance
Source: Nutrients. 2020 Apr 22;12(4):1161. doi: 10.3390/nu12041161 (PMC7231187; doi:10.3390/nu12041161)
Supplement: Supplementary file 1 [file nutrients-12-01161-s001.pdf]

TableS1. Effects on energy expenditure and hypothesized mechanisms to induce weight loss

Food	Main active compound(s)	Effect(s) on EE	Mechanisms of action	References
Green coffee (Coffea canephora, Coffea Arabica)<br>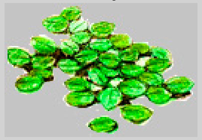	Caffeine, chlorogenic acid	Enhanced thermogenesis<br>Increased REE<br>Browning of WAT<br>Increased fatty acids oxidation	Up-regulation of UCP1 and BAT-selective regulatory genes<br>Activation of cAMP<br>β2- and β3-adrenergic receptors stimulation<br>Up-regulation of AMPK<br>Antagonism of TRPV-4	[55]****<br>[57]**<br>[58]****<br>[59]* [60]<br>[61]**** [63]*<br>[62]* [64]**
Green Tea (Camellia sinensis)<br>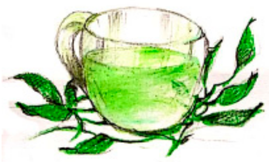	Catechins, caffeine	Enhanced thermogenesis<br>Increased EE<br>Increased fat oxidation	Inhibition of phosphodiesterase enzymes<br>Up-regulation of the gene expressions of UCPs<br>Inhibition of norepinephrine degradation with increased SNS activity<br>Stimulation of substrates in Cori cycle and free fatty acid-triglyceride cycle<br>Activation of AMPK<br>Increased glycolysis and ATP turnover	[68]* [69]*<br>[70] [71]*<br>[72]* [73]**<br>[74]**/***<br>[75]*** [76]*
**Cocoa and dark chocolate**<br>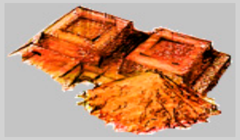	Caffeine, theobromine, polyphenols	Increased thermogenesis in liver and in WAT<br>Increased mitochondrial biogenesis<br>Fatty acid oxidation	Increased expression of thermogenic genes and release of free-fatty acids<br>Induced expression of UCPs, PGC-1α and AMPKα<br>Adenosine receptor blockage<br>Stimulation of basal and noradrenaline-induced lipolysis<br>Activation of AMPK	[79]** [80]*<br>[81] [82]****<br>[83] [84]*<br>[85]* [86] [87]<br>[88] [90]* [91]<br>[92]*
Yerba Mate (Ilex Paraguariensis)<br>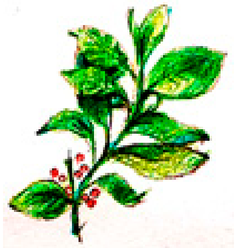	Polyphenols, alkaloids, triterpenoids, flavonoids, chlorogenic acid	Increased REE<br>Increased fatty acids oxidation<br>Inhibition of adipogenesis<br>Increased AEE<br>Enhanced thermogenesis in WAT<br>Reduced fatty acids synthesis in WAT	Up-regulation of AMPK,<br>Down-regulation of genes *Creb-1* and *C/EBPa*<br>Preferential use of fatty acids as energy substrate<br>Activation of mitochondrial genesis<br>expression of UCPs	[63]* [95]*<br>[96] [97]**<br>[98]** [99]<br>[100] [101]

Bitter Orange (Citrus Aurantium)<br>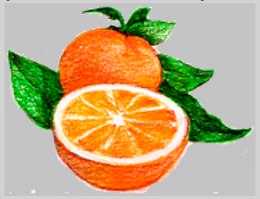	Alkaloids (synephrine, octopamine)<br><br>Flavonoids (hesperidin, naringin, limonene, tangaretin	Enhanced thermogenesis in WAT<br>reduced fatty acids synthesis in WAT<br>Increased REE and DIT<br>Increased mitochondrial biogenesis	Enhanced expression of UCPs	[104]* [105]* [106]* [107]** [108]** [109]* [111] [112]
**Ginger (*Zingiber officinale*)**<br>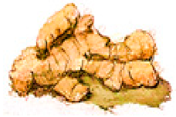	Zingerone	Enhanced thermogenesis<br><br>Increased DIT<br>Enhanced BAT function<br>Activated WAT browning<br>Increased mitochondrial biogenesis<br>Increased fatty acid catabolism	Activation of AMPK signaling in WAT<br>Activation of TRPV1 channels (adrenaline releasing)<br>Activation of the sirtuin-1 (SIRT1)/AMPK/ PGC-1α pathways<br>Transcriptional regulation of energy metabolizing proteins<br><br><br><br>Activation of the PPARδ pathway	[117] [118] [119] [120] [121 [122]
**Turmeric, (*Curcuma longa*)**<br>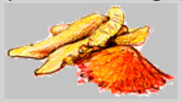	Curcumin, turmerone	Enhanced thermogenesis<br>Enhanced lipolysis	Activation of AMPK with increased UCP1 expression<br>Increased mitochondrial biogenesis<br>Increased expression of β3- adrenoreceptors (and norepinephrine plasma levels)<br>Upregulation of the cAMP/PKA/CREB pathway	[129] [130] [132]
**Cinnamon**<br>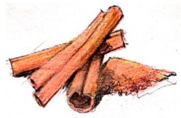	Cinnamaldehyde	Enhanced thermogenesis<br><br><br><br>Increased EE	Increased expression of UCP1 in BAT<br>Activation of phospho-AMPK<br>Regulation of cAMP dependent protein kinase/p38 MAPK-dependent pathway<br>Activation of the TRPA1 (adrenalin secretion)	[136] [137]** [138]** [140] [142] [143] [144] [145]
**Chilli pepper (*Capsicum species*)**<br>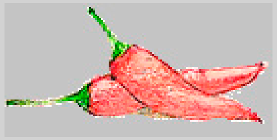	Capsaicinoids (capsaicin and dihydro-capsaicin), capsinoids (capsiate, dihydro-capsiate, nordihydro-capsiate)	Enhanced thermogenesis<br>enhanced EE<br>Activation and recruitment of BAT<br>Increased EE<br>decrease RQ<br>reduction of the decrease in DIT and REE during hypocaloric diet	Up-regulation of the expression of SIRT1 and PGC-1α with increased expression of UCP1 and bone morphogenetic protein 8b<br>Stimulation of the SNS and the catecholamine secretion from adrenal gland<br>Activation of TRPV1 and stimulation of the SNS	[19]* [137]** [151]* [152]* [153]* [154]* [155]** [156] [157]*

Garcinia Cambogia<br>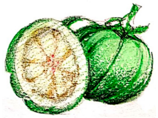	Hydroxy-citric acid,<br>anthones,<br>benzophenones	Increased BEE<br>increased fat oxidation<br>decreased *de novo* lipogenesis	Activation of the adiponectin-AMPK signalling pathway<br>Regulation of thyroid hormone levels<br>Down-regulation of ATP-citrate lyase	[159]* [160]*<br>[161]* [162]**<br>[163]***<br>[164]*** [166]<br>[167]
**Guarana *(Paullinia cupana)***<br>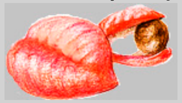	Caffeine,<br>polyphenols,<br>theobromine,<br>theophylline	Increased thermogenesis<br>Increased BEE<br>Inhibited adipogenesis	Induced BAT expansion<br>Increased mitochondrial biogenesis<br>Enhanced UCP1expression<br>AMPK activation	[176]**<br>[177]*** [178]<br>[179]
**Brassicaceae (or Cruciferae)**<br>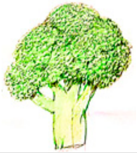	Glucoraphanin (hydrolyzed to the biologically active sulforaphane)	Enhanced thermogenesis<br>increased mitochondrial biogenesis function<br>enhanced EE<br>browning of WAT	Up-regulation of the Nrf2/SIRT1/PGC-1α signalling<br>Enhanced UCP1 expression in beige adipocytes	[182] [183]<br>[185] [186]<br>[187] [188]
**Nuts**<br>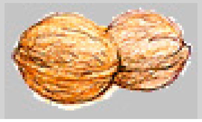	Flavonoids,<br>MUFA,<br>PUFA,<br>Vitamins	Increased DIT<br>Increased BEE		[24]* [51]**<br>[192]*<br>[193]***<br>[194]**
**Apple cider vinegar**<br>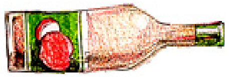	Acetic acid	Enhanced thermogenesis and fatty acids oxidation	Regulation of α2-AMPK/PPARα-mediated pathway with up-regulation of the expression of genes acetyl-CoA oxidase, carnitine palmitoyl transferase-1 and UCP2	[198]
**Spirulina (*Arthrospira platensis and maxima*)**<br>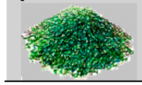		No data on EE		

*Review including trials on humans with acute and/or chronic administration of product(s)
** Trial on humans after acute administration of product(s)
*** Trial on humans after chronic administration of product(s)
**** Observational study on humans after acute administration of product(s)

Abbreviations: AEE: activity-induced energy expenditure, AMPK: adenosine monophosphate activated protein kinase, ATP: adenosine triphosphate, BAT: brown adipose tissue, BEE: basal energy expenditure, cAMP: cyclic adenosine monophosphate, CREB: cAMP response element-binding protein , DIT: diet-induced thermogenesis, EE: energy expenditure, MAPK: mitogen-activated protein kinase, MUFA: monounsaturated fatty acids, Nrf2: nuclear factor erythroid 2-related factor 2 , PGC-1α: PPARγ coactivator-1 α, PPAR: proliferator-activated receptor, PKA: protein kinase A, PUFA: polyunsaturated fatty acids, REE: resting energy expenditure, RQ: respiratory quotient, SIRT1: sirtuin 1, SNS: sympathetic nervous system, TRPA1: Transient Receptor Potential Ankyrin 1, TRPV: transient receptor potential vanilloid, UCP: uncoupling protein, WAT: white adipose tissue.
